# Channeling Community Contributions to Scientific Software: A Sprint Experience

**DOI:** 10.5334/jors.96

**Published:** 2016-07-19

**Authors:** Michael R. Crusoe, C. Titus Brown

**Affiliations:** 1 Microbiology and Molecular Genetics, Michigan State University, East Lansing, MI, USA; 2 Computer Science and Engineering, Michigan State University, East Lansing, MI, USA

**Keywords:** code sprint, onboarding, hackathon, khmer, bioinformatics

## Abstract

In 2014, the khmer software project participated in a two-day global sprint coordinated by the Mozilla Science Lab. We offered a mentored experience in contributing to a scientific software project for anyone who was interested. We provided entry-level tasks and worked with contributors as they worked through our development process. The experience was successful on both a social and a technical level, bringing in 13 contributions from 9 new contributors and validating our development process. In this experience paper we describe the sprint preparation and process, relate anecdotal experiences, and draw conclusions about what other projects could do to enable a similar outcome. The khmer software is developed openly at http://github.com/dib-lab/khmer/.

## (1) Introduction

Sustainable development of scientific software inevitably depends on following good software development practices. However, even rudimentary development practices such as version control and testing are rarely a formal part of scientific training. One way to learn these practices is to participate in an open source project, which often provide a path for new contributors to get involved. Open source *scientific* software projects can go further by providing scientists the opportunity to work on a science-focused project.

In July 2014, Mozilla Science Lab (MSL) ran a two-day global “sprint” for a wide variety of software projects. As part of this sprint the khmer project offered a mentored software contribution experience. The khmer project is a bioinformatics library developed primarily at Michigan State University, and it uses many open source software development practices [3,4]. These practices include open development and code review on GitHub using a workflow called GitHub Flow [2], the maintenance of a large suite of unit and functional tests, continuous integration, formal release testing, and semantic versioning. The two authors of this paper, MRC and CTB, are respectively the lead software engineer and the principle investigator on the NIH grant that funds MRC and khmer development.

The basic motivating principle of many scientific hackathons and datathons is to gather a group of people together to work in a focused, coherent way on one or more projects (reviewed in [5]). Our primary goal for participating in the MSL sprint was not to make significant progress on the technical aspects of the code, but rather to train scientists in version control and code review and improve our documentation and processes so as to lower the barriers to entry for new developers to our project. In this case, we took advantage of the distributed nature of the Mozilla event to recruit participants globally, with no travel required. We also decided not to focus on expert scientists or developers, but rather on participants who were interested in but perhaps not engaged in open science or open source practices.

We had several concerns when organizing our part of the sprint. We were uncertain how to target the list of issues for an unknown number of developers with a potentially wide range of development experience. We were also concerned that our development process involved too many steps for new developers to work through. Finally, we were unsure of whether this would be an effective use of our time. Despite these reservations, we participated in the sprint because the sprint would be an opportunity to use new developers to expose problems in our documentation and software. We also took advantage of the sprint to ask local lab members to go through the full development cycle themselves.

The global sprint was organized as follows: each physical location was asked to provide directions for attendees, along with coffee, Internet connections, and a video wall. Mozilla then connected these into a global video wall, and also provided a central IRC channel for the sprint. The goal of the sprint was to provide a supportive environment in which to collaborate on open science projects, encourage contributions from new people, and introduce new people to a variety of projects. Standard work hours, breaks for lunch and snacks, and an emphasis on acknowledging a diversity of contributions were all part of the setup. There was also a pan-site Code of Conduct provided, which was widely advertised and may have led to more significant buy-in from certain communities.

In the end, the two-day sprint was a modest success technically, and a big success socially. We merged 13 contributions from 9 distinct contributors into the master development branch; we solved a previously unappreciated installation problem with our software; and we revamped our development documentation to include a detailed guide to getting started. Overall, we felt that the sprint was a very useful investment of our time and energy and are looking forward to future sprints.

Below, we describe the pre-sprint preparation, the sprint itself, and the post-sprint outcomes. We then provide some concluding thoughts.

## (2) Pre-sprint preparation

We announced the sprint in a blog post [1], broadcast the blog post via Twitter, evangelized it at two conferences, and entered it into the Mozilla Science Lab project list. We then provided an issue on our GitHub issue tracker for people to subscribe to for updates. This provided a more specific notification channel than a mailing list for us to use in informing contributors of our plans, and also ensured that interested parties already had a GitHub account.

We next designated a set of issues with a “low-hanging fruit” tag. These issues were chosen (or in some cases designed) to be entry-level: they required no biology or bioinformatics knowledge, and no prior experience with the khmer project was needed. The issues targeted a range of Python, C++, and documentation changes. For example, one issue involved replacing a C++ stdlib exception with a khmer specific one, while another issue required copying an existing test and making a minor modification.

Finally, we wrote a detailed walk-through for new contributors.^1^ This walk-through assumed some prior command-line expertise and basic familiarity with git, but otherwise required no particular familiarity with GitHub, the GitHub Flow process, khmer installation, or anything specific to khmer development. Crucially, much of this workflow was written to be copy-paste at the command line, which avoided the burdensome requirement for inexperienced developers to compose many new commands.

The workflow covered twenty five distinct steps and included forking a copy of khmer on GitHub, cloning it locally, building khmer, running the tests, claiming an issue, making changes and committing them, verifying the changes by running the tests again, pushing back to GitHub, and going through continuous integration and code review.

## (3) During the sprint

The sprint ran for two days, July 22 and 23, from 9am to 5pm EST (Michigan local time). We merged 13 pull requests that were both started and finished during this period, contributed by five remote and four local users. Our in-person contributors included someone from industry who took vacation days to attend the sprint; several people loosely affiliated with the lab but who had not previously contributed to the codebase; and another member of the MSU community who was unaffiliated with our lab.

### Activities

During the sprint, we interacted with participants, revised the central sprint issue, and updated our documentation regularly in response to problems. CTB primarily focused on updating the documentation while MRC primarily interacted with remote and local developers.

We enforced a requirement that each contributor completed all the items on our development checklist, just like any other contributor. However, MRC found that it was difficult to balance detailed code review with the many different demands on his time as multiple contributors updated issues, encountered problems, and had questions. This was an area where more code reviewers would be needed to scale.

### Communication and Environment

Throughout the sprint, Internet Relay Chat (IRC) provided a realtime venue for private and group chat that supported our issue-driven process. We had little direct interaction over video, because we were a small part of the larger Mozilla Science Lab sprint. However, the sense of community and cooperation was greatly enhanced by the presence of the always-on video wall.

The environment was friendly and relaxed, with a welcoming physical environment and good community feeling. We shared the sprint space with a Data Carpentry sprint as well, which helped build community feeling. We used Twitter to announce first-time contributors on the first day, and CTB provided a running commentary as issues came up and were addressed.

### Issues and problems

The most important issue that surfaced during the sprint was that our test running commands simply didn't work for many. We had included some installation commands in the `make test' command that depended on certain versions of Python build infrastructure. Many of our sprint participants ran into this problem in the first two hours of the sprint, leading us to debug and change the installation commands live, and then update the instructions.

We also found that many contributors did not reliably follow the detailed instructions. This was not surprising – 25 steps is quite a lot! – but we couldn't find a way to simplify our workflow, either. However, because we were providing support in real time, we could almost always give useful feedback to help participants discover which steps they had missed and correct them. A longer feedback cycle might have led to many orphaned pull requests as contributors gave up on our workflow.

## (4) Post-sprint feedback and actions

We had 9 participants who both started and finished a total of 13 pull requests during the sprint; five were remote, and four were local.

None of our nine participants had experienced GitHub Flow before, and most had no prior exposure to testing or code review. Several participants expressed enthusiasm for having gone through the process. A further 3 participants are still working on finishing pull requests started during the sprint.

We are further revising the documentation after post-sprint review, to better link different sections and refine sections that were updated hastily during the sprint.

## (5) Discussion and concluding thoughts

We felt that the most valuable part of the sprint was in setting aside this focused time for in-lab problem solving and collaboration. Most of the khmer developers were in the room together and when a problem needed to be discussed (e.g. the installation problem) it was easy to hold an impromptu meeting. This is different from our usual lab development process which is largely asynchronous.

The rapid, systematic review, improvement and testing of documentation was tremendously valuable; having put 10 or more participants through our “getting started” documentation means that we are now certain that the instructions work! However, more observation of inexperienced contributors will undoubtedly lead to areas where can optimize the documentation for first-time participants.

In the long term, we do not expect many, or perhaps any, of the sprint participants to continue developing on khmer. None of the participants external to the lab worked in our subfield of biology, and khmer itself has a fairly narrow set of functionality. However, we can guess that because of the improved documentation, khmer will now be better able to attract contributions from developers who *are* interested in longer-term engagement with the project.

We do hope that the sprint participants will use their new experience with GitHub, distributed version control, and remote development to contribute to other open source projects. We plan to query their GitHub activity on public projects to see if there is additional engagement in the months to come.

The presence of existing process and infrastructure let us work with new contributors more easily than we would have been able to a year ago: they got more done. In turns this meant that we could leverage their contributions more easily: we gained more from what they did. Process documentation, issue tracking, tests, reliable build and test instructions, and mechanisms for support were all important. The up-front organization specific to our sprint was minimal, because we already had many existing resources. Moreover, the getting-started guide and the low-hanging-fruit issues provide an excellent entree into our software project that remains after the sprint.

It was important to have two active, dedicated participants so that specific issues (pull requests and technical support) as well as meta-issues (documentation and communication) could be handled. We believe the process would not have scaled much beyond 2–3 simultaneous participants without an additional khmer developer, which could be a bottleneck for projects; perhaps our next khmer sprint will focus on training new code reviewers!

The biggest unresolved challenge is how to more effectively walk participants through their first contribution. While 25 steps may seem overly complex, each step is an important part of the development cycle; experienced software developers may elide many of the steps mentally (“of *course* I run the tests after each commit!”) but they are all necessary. This complexity illuminates the challenge facing scientists who want to learn basic software development practices: each development practice (e.g. using version control, or testing, or code review) requires that many different steps be executed in combination. Our experience from the sprint suggests that participants can be taught to execute these steps fairly easily, if sufficient time and support is provided.

Future revisions to our on-boarding documentation could simplify the documentation in a few ways by eliminating optional steps (e.g. our current documentation provides instructions for using ccache). Apart from that, we could more formally study the “first-time contributor” workflow by working with people as they go through it, to see where mistakes are commonly made. We are wary of oversimplifying, however, because simplifying further could result in increased maintenance burden on our part, and also diminish the ability of people to transition from our project (which uses a fairly standard GitHub-flow based workflow [2]) to others.

We are looking forward to future sprints and would like to involve more scientific software development groups in teaching others about their development workflows.

## Footnotes

1 http://khmer.readthedocs.org/en/docs-hackathon/dev/getting-started.html
